# The genome of the American dog tick (*Dermacentor variabilis*)

**DOI:** 10.1093/g3journal/jkaf130

**Published:** 2025-06-09

**Authors:** Jacob Cassens, Matt Villalta, Saul Aguirre, Lauren Ecklund, Trek Stenger, Idil Abdi, Sree Venigalla, Elizabeth Shiffman, Kristen Bastug, Beth K Thielen, Christopher Faulk

**Affiliations:** Division of Environmental Health Sciences, School of Public Health, University of Minnesota Twin Cities, Minneapolis, MN 55455, USA; ANSC 8520 Students, University of Minnesota Twin Cities, Minneapolis, MN 55455, USA; ANSC 8520 Students, University of Minnesota Twin Cities, Minneapolis, MN 55455, USA; ANSC 8520 Students, University of Minnesota Twin Cities, Minneapolis, MN 55455, USA; ANSC 8520 Students, University of Minnesota Twin Cities, Minneapolis, MN 55455, USA; ANSC 8520 Students, University of Minnesota Twin Cities, Minneapolis, MN 55455, USA; ANSC 8520 Students, University of Minnesota Twin Cities, Minneapolis, MN 55455, USA; Minnesota Department of Health, Division of Infectious Disease Epidemiology, Prevention and Control, Saint Paul, MN 55164, USA; Division of Pediatric Infectious Diseases, Department of Pediatrics, Medical School, University of Minnesota Twin Cities, Minneapolis, MN 55455, USA; Division of Pediatric Infectious Diseases, Department of Pediatrics, Medical School, University of Minnesota Twin Cities, Minneapolis, MN 55455, USA; Department of Animal Science, College of Food, Agricultural and Natural Resource Sciences, University of Minnesota Twin Cities, Saint Paul, MN 55108, USA

**Keywords:** tick, nanopore, genome assembly, symbionts, epigenetics

## Abstract

The American dog tick (*Dermacentor variabilis*) is a vector of zoonotic pathogens in North America that poses emerging threats to public health. Despite its medical importance, genomic resources for *D. variabilis* remain scarce. Leveraging long-read nanopore sequencing, we generated a high-quality genome assembly for *D. variabilis* with a final size of 2.15 Gb, an N50 of 445 kb, and a benchmarking universal single-copy ortholog (BUSCO) completeness score of 95.2%. Comparative BUSCO analyses revealed fewer duplicate genes in our assembly than in other *Dermacentor* genomes, indicating improved haplotype resolution. The mitochondrial genome, assembled as a single circular contig, clustered monophyletically with *D. variabilis* isolates from the Upper Midwest, corroborating regional phylogenetic relationships. Repetitive element analysis identified 61% of the genome as repetitive, dominated by long interspersed nuclear elements and long terminal repeat elements, with 24% remaining unclassified, underscoring the need for further exploration of transposable elements in tick genomes. Gene annotation predicted 21,722 putative genes, achieving a protein BUSCO completeness of 80.88%. Additionally, genome-wide methylation analysis revealed 9.9% global 5mC methylation, providing the first insights into epigenetic modifications in *D. variabilis*. Further, nanopore sequencing detected *Rickettsia montanensis* and a nonpathogenic *Francisella*-like endosymbiont. These findings expand our understanding of tick genomics and epigenetics, offering valuable resources for comparative studies and evolutionary analyses.

## Introduction

*Dermacentor variabilis*, also known as the American dog tick or wood tick, is an obligate blood-feeding ectoparasite found throughout North America, first described over 200 years ago (Lado *et al*. 2019). The American dog tick has 3 post-embryonic life stages (larva, nymph, adult), each requiring a blood meal for development and reproduction. Each life stage provides an opportunity to acquire a blood meal, and *D. variabilis* is a 3-host tick that can feed on a different host species at each life stage. The immature life stages commonly parasitize smaller mammals, such as rodents, while adults primarily feed on larger animals, including dogs and deer (Duncan *et al*. 2021). This blood-feeding strategy facilitates the enzootic maintenance of tick-borne pathogens. The American dog tick is a vector for human pathogenic Rocky Mountain Spotted Fever (*Rickettsia rickettsii*) and Tularemia (*Francisella tularensis*). It has also been found infected with other bacterial (*Anaplasma marginale*, *Ehrlichia* spp.), protozoal (*Toxoplasma gondii*, *Cytauxzoon felis*, *Babesia* spp.), and viral (Powassan) pathogens, although its vector competency for these agents is unknown (Shock *et al*. 2014; Ben-Harari 2019; Whitten *et al*. 2019; Duncan *et al*. 2021; Steiert and Gilfoy 2002; Reichard *et al*. 2021; Hart *et al*. 2023). Additionally, *D. variabilis* exhibits aggressive host-seeking behavior and is most active during times of the year with increased human outdoor activity (e.g. April–September), making it one of the most frequently encountered hard ticks in the United States (Duncan *et al*. 2021). Although pathogen prevalence in natural populations remains low (Eisen *et al*. 2017), expanding genomic resources for hard ticks like *D. variabilis* is critical to generating fundamental insights into tick biology and addressing global zoonotic threats.

Ticks present unique challenges to genomic research due to their large genome sizes, high proportion of repetitive elements, and complex life cycles. Genomic studies on *D. variabilis* and other ticks have been limited yet are critical as ticks continue to expand their geographic ranges and pose growing threats to public health. The American dog tick belongs to the class Arachnida, order Ixodida, family Ixodidae, and group Metastriata. Metastriate ticks comprise 13 genera with over 450 species, representing a derived lineage relative to the basal Prostriata (Mans *et al*. 2016). Currently, only 10 unique genome assemblies for metastriate ticks are available on NCBI, and these resources have proved valuable for investigating tick biology and evolution. For example, comparative studies of metastriate and prostriate genomes in Europe and Asia have highlighted lineage-specific evolutionary trajectories of tick–host interactions, tick immunity, and ecological adaptations (Jia *et al*. 2020; Cerqueira De Araujo *et al*. 2025).

The recent advancements in long-read sequencing technologies, such as Oxford Nanopore Technologies (ONT) and Pacific Biosciences (PacBio), have revolutionized tick genomics by overcoming challenges associated with assembling large and repetitive genomes. Our study contributes to this growing field by presenting the first whole-genome assembly for *D. variabilis*. This high-quality genome assembly provides a foundational resource for comparative genomic analyses, population genetics, and evolutionary studies. Further, as obligate blood feeders, ticks are a repository for exogenous DNA—previous blood meals, endosymbionts, and pathogens—that is recoverable from a single sample using native genomic sequencing. We demonstrate the utility of long-read sequencing technologies in differentiating tick DNA from pathogenic and nonpathogenic DNA while broadening our understanding of the genetic diversity, evolutionary history, and adaptive strategies of medically significant arthropods. Developing genomic resources for *D. variabilis* enhances the ability to identify novel targets for transgenic techniques and pest management strategies, aiding efforts to mitigate the impact of hard ticks on public health.

## Methods

### Sample collection

Adult female *D. variabilis* ticks found attached to a companion canine after an outdoor walk in central Minnesota were removed and stored in 100% ethanol. Ticks were morphologically identified using the taxonomic key from Yunker et al. (1986). Ticks were transferred to Zymo DNA/RNA shield for 1 h prior to DNA extraction and library preparation.

### DNA extraction and sequencing

Genomic DNA was extracted from 10 adult female ticks using a MagAttract Blood DNA/RNA Kit (Qiagen, Venlo, Netherlands) according to the manufacturer's instructions. Fragment size was assessed on an agarose gel. Size selection for fragments >10 kb was attempted by treatment with a short fragment eliminator XS kit from PacBio. However, insufficient DNA was recovered for sequencing, necessitating the sequencing of short fragments only. Sequencing was performed on a P2 Solo instrument (ONT) using a single PromethION R10.4.1 flow cell. Data were collected using MinKNOW v23.07.12 in the 5 kHz condition.

Raw nanopore data in pod5 format were re-basecalled using dorado v0.8.2 (https://github.com/nanoporetech/dorado) with model dna_r10.4.1_e8.2_400bps_sup@v5.0.0, and modifications called simultaneously with the flag “--modified-bases 5mC_5hmC”. Read quality was assessed using the Nanoq package (https://github.com/esteinig/nanoq).

### Genome assembly

The genome was de novo assembled using Flye v2.9 on a high memory (2 Tb) node due to the expected assembly size and our read depth (https://github.com/fenderglass/Flye). The resulting draft assembly was processed with the NCBI Foreign Contaminant Screen program to remove vector and adapter sequences (https://github.com/ncbi/fcs). Duplicate contigs and overlaps were removed with Purge_dups v1.2.6 (https://github.com/dfguan/purge_dups). Gaps were filled in the assembly by using the ntLink v1.3.11 scaffolding program with 6 rounds of linking and gap-filling (https://github.com/bcgsc/ntLink). The resulting scaffolded assembly was split back into contigs at all remaining unfilled gaps, and “n” bases were removed, creating a more contiguous contig-level assembly. ntLink includes both a misassembly correction and gap purging step that may fragment contigs due to structural errors, poorly supported reads, or ambiguous sequences, leading to corresponding changes in the number of contigs and contig N50. Despite this, ntLink provides another layer of reliability to genome scaffolding and assembly.

The genome was decontaminated using Blobtoolkit v4.4.0 (https://blobtoolkit.genomehubs.org). First, the contigs were annotated using locally installed NCBI BLAST with the “core_nt” database. Second, contig depth was calculated using samtools coverage. Finally, the Blobtools database was generated and annotations analyzed in a browser. Hits assigned to “arthropoda” “no-hit” were kept and all other contigs removed. We also removed any contigs with less than 10× or greater than 1,000× depth. The resulting assembly was designated as the reference. Results from the Blobtools analysis are available in Supplementary File 4.

After each assembly iteration, the completeness of draft assemblies was evaluated using the standard benchmarking universal single-copy ortholog (BUSCO) count with the Arachnida lineage (Simão *et al*. 2015). We used the compleasm package (https://github.com/huangnengCSU/compleasm; Huang and Li, 2023) to generate completeness scores as it offers a faster, more accurate implementation of BUSCO. We combined the single and duplicate counts from the compleasm scores to provide a direct comparison to BUSCO's “complete” value. Genome summary statistics, such as N50 and L50, were calculated using assembly-stats (https://github.com/sanger-pathogens/assembly-stats). The quality of each draft assembly was assessed by the read N50 (i.e. contig length at which 50% of the assembled genome is contained in longer contigs), read L50 (i.e. number of contigs whose cumulative length covers half the assembled genome), and the count of BUSCOs present in the assembly. Genome contiguity was visualized using the QUAST 5.3.0 package (https://quast.sourceforge.net).

### Nuclear phylogeny

To infer nuclear phylogenetic relationships, we compared our *D. variabilis* genome assembly to other available Metastriate tick nuclear genomes, using the Prostriate tick *Ixodes scapularis* as an outgroup. Amino acid sequence files for related tick species were downloaded from NCBI. We used OrthoFinder v2.5.5 to identify orthologous genes by extracting the longest transcript isoform per gene and conducting gene-tree based species tree inference (Emms and Kelly 2019). The resulting species tree was visualized using FigTree v1.4.4 (https://github.com/rambaut/figtree/releases).

### Mitochondrial assembly

The mitogenome was extracted and characterized from the reads using MitoHiFi v3.2.2 (https://github.com/marcelauliano/MitoHiFi). MitoHiFi identifies putative mitogenomic contigs by comparing them to reference mitogenomes from related species (Uliano-Silva *et al*. 2023). We manually specified the reference *Dermacentor silvarum* mitogenome for comparison (NC_026552.1). Default parameters were used with specifications for the invertebrate mitochondrial code. MitoHiFi identified, circularized, and annotated a single putative contig. The assembled mitogenome was visualized using OGDRAW v1.3.1 (Greiner *et al*. 2019).

### Mitochondrial phylogeny

Our *D. variabilis* mitogenome was compared against others available in NCBI along with related species to build a phylogeny using MAFFT v7.5 for alignment (Katoh *et al*. 2019). Trees were generated and bootstrapped using Iqtree2 v2.3.6 (https://github.com/iqtree/iqtree2) and visualized using FigTree v1.4.4 (https://github.com/rambaut/figtree/releases).

### Repeats

Initial repeat content identification was performed using RepeatMasker v4.1.4 (https://www.repeatmasker.org/) with the complete Dfam library v3.6 (https://www.dfam.org/home) as described by Flynn *et al*. (2020) and Storer *et al*. (2021). RepeatMasker masked a low proportion of the genome, indicating that much of the genome's repetitive content is not annotated in the public database. Thus, we compiled a custom database of repetitive element content using RepeatModeler2 (https://github.com/Dfam-consortium/RepeatModeler). We specified the entire *D.* variabilis genome assembled herein to RepeatModeler for database building, We then re-ran RepeatMasker with the custom *D. variabilis* database (via RepeatModeler2) built with RepeatModeler to identify the complete repetitive element content in our assembly (Flynn *et al*. 2020). Detailed computational pipelines and code are available in Supplementary File 1.

### Methylation

Nanopore sequencing enables the direct detection of base modifications, such as 5-methylcytosine (5mC) and 5-hydroxymethylcytosine (5hmC), by measuring ionic current changes as nucleotides pass through nanopores on a flow cell. These current changes, determined by the molecular weights of modified vs unmodified bases, allow for methylation detection without requiring additional molecular preparation. The global methylation and hydroxymethylation (5mC and 5hmC) at CpG dinucleotides were determined using the modified base information stored in the basecalled BAM files. Modified BAM files were concatenated and mapped back to our draft assembly using Minimap2 (https://github.com/lh3/minimap2). The modified mapped BAM was converted to bedMethyl format using modkit v0.3.4 (https://github.com/nanoporetech/modkit), and global 5mC and 5hmC percentages were summarized using AWK (https://www.gnu.org/software/gawk/manual/gawk.html#Manual-History).

### Gene annotation

Gene Model Mapper (GeMoMa) v1.9 (Keilwagen *et al*. 2019) was used for homology-based gene prediction using transcripts from *I. scapularis* (ASM1692078v2) as a reference. BUSCO was executed in protein mode with the arachnida_odb10 database to assess prediction accuracy and completeness. Information on gene annotation features are available in Supplementary File 5.

### Symbiont detection

The total reads were aligned in succession to reference genomes from *Francisella* species (GCF_001275365.1, GCF_003347135.1, GCF_000833355.1) and *R. rickettsii* (GCF_000018225.1). Breadth and depth were calculated with samtools. Data were visualized with BAMstats v1.25 (https://bamstats.sourceforge.net).

## Results and discussion

### Assembly

We generated a total of 83.9 Gb of long-read sequence data for the American dog tick (*D. variabilis*) with an N50 of 3.6 kb and a mean quality of Q19.1. The relatively short N50 was driven by the difficulty in extracting high molecular weight DNA from adult ticks. Our initial Flye run yielded a draft assembly of 2.5 Gb organized into 109,188 contigs with an N50 of 118 kb (Table 1). The initial assembly was cleaned of contaminant sequences (e.g. vector, adapter, and symbiont), purged of duplicate contigs, and gaps were filled to increase contiguity. Our final assembly was 2.15 Gb consisting of 21,695 contigs with an N50 of 445 kb, achieving 36.6× coverage.

**Table 1.** Genome contiguity and quality statistics for each iterative draft assembly for the reported *Dermacentor variabilis* genome.

Assembly	Size (bp)	Contigs	N50 (bp)	L50	BUSCO^a^ complete (%)	BUSCO single (%)	BUSCO duplicate (%)	Gaps (*n*)
Initial assembly (Flye)	2,509,290,473	109,188	118,641	4,871	92.26	90.56	1.70	0
Purged (purge_dups)	2,192,205,988	56,222	146,707	3,707	95.20	93.97	1.23	0
ntLinked	2,177,726,303	27,881	438,836	1,304	95.20	93.97	1.23	89
Purged (nogaps)	2,177,300,621	27,970	435,334	1,318	95.20	93.97	1.23	0
Decontaminated	2,148,583,136	21,695	445,025	1,285	95.20	93.97	1.23	0

^a^BUSCOs calculated with the compleasm tool.

**Fig. 1.**
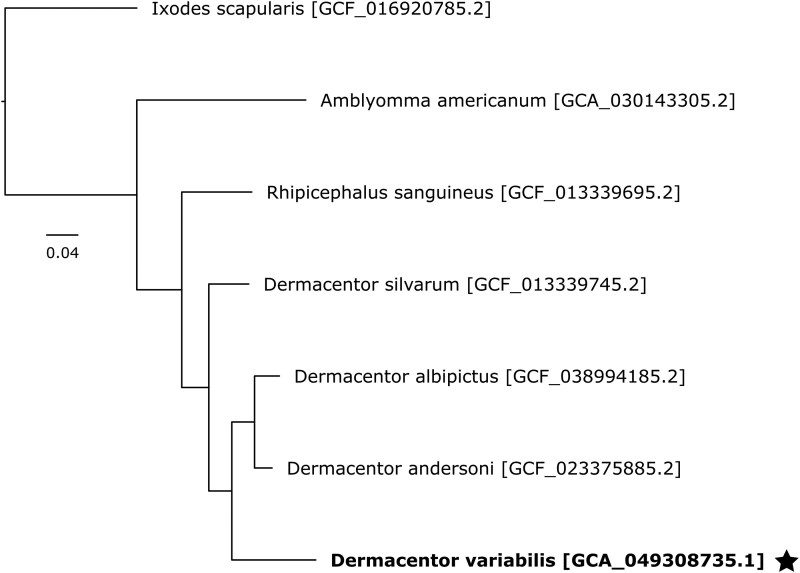
Phylogenetic relationships between nuclear genomes of *Dermacentor*, *Amblyomma*, *Rhipicephalus*, and *Ixodes* species were reconstructed using gene orthology inference with OrthoFinder. Phylogenies were created using the longest variant transcript per gene.

Our final assembled genome had a BUSCO completeness score of 95.2%, comparable to 3 other published *Dermacentor* genomes (Table 2). BUSCO completeness scores can be deconstructed into single- and duplicate-copy percentages that provide more nuanced information about genome assembly quality. Single-copy genes suggest high-quality assembly, while duplicate-copy genes may indicate assembly errors. The other *Dermacentor* genomes had BUSCO scores over 97% with 2–4% duplicate-copy genes, while ours had a lower completeness score but fewer duplicates (<1% after purging). This is notably lower than the >2% duplicates in *Dermacentor albipictus* and *Dermacentor andersoni* and up to 4% in *D. silvarum*, suggesting potential uncollapsed haplotigs in those assemblies. Our phylogenetic analysis of tick nuclear genomes revealed that *D. albipictus and D. andersoni* are more closely related to each other than to *D. variabilis*, yet all 3 form an American *Dermacentor* clade distinct from *D. silvarum*, which occurs in Asia. Further, the *Dermacentor* species cluster to the exclusion of the other Metastriate ticks, *Rhipicephalus sanguineus* and *Amblyomma americanum*. This Metastriate clade is clearly distinct from the Prostriate tick *I. scapularis*, which serves as an outgroup (Fig. 1).

**Table 2.** BUSCO scores for the reported *Dermacentor variabilis* genome assembly and the other publicly available *Dermacentor* genome assemblies.

Species	Build	BUSCO^a^ complete (%)	BUSCO single (%)	BUSCO duplicate (%)
*Dermacentor variabilis*	This report	95.20	93.97	1.23
*Dermacentor albipictus*	USDA_Dalb1.pri	97.92	95.60	2.32
*Dermacentor silvarum*	BIME_Dsil_1.4	97.41	93.39	4.02
*Dermacentor andersoni*	qqDerAnde1.2	97.65	95.57^a^	2.08

^a^BUSCOs calculated with the compleasm tool.

Tick genomes are challenging to assemble due to their large size, complexity, and high proportion of repetitive elements. Short-read sequencing technologies often fail to resolve long repetitive sequences, limiting the generation of contiguous tick genomes. Long-read sequencing technologies, such as ONT and PacBio, can span these repetitive sequences, enabling more complete assemblies. Advancements in third-generation sequencing technologies over the past decade have led to a surge in assembled tick genomes, and our assembly contributes to this growing resource, enhancing efforts to understand tick biology. However, our assembly was generated from a pool of *D. variabilis* tick, which introduces certain limitations. Pooling restricted our ability to assess individual-level genetic variation, constrained the contiguity of the assembly, and excluded male-derived material, preventing the comparison of sex chromosomes. These factors likely contributed to the fragmented nature of the final assembly, which lacks large, contiguous scaffolds.

### Mitochondrial structure and phylogeny

**Fig. 2.**
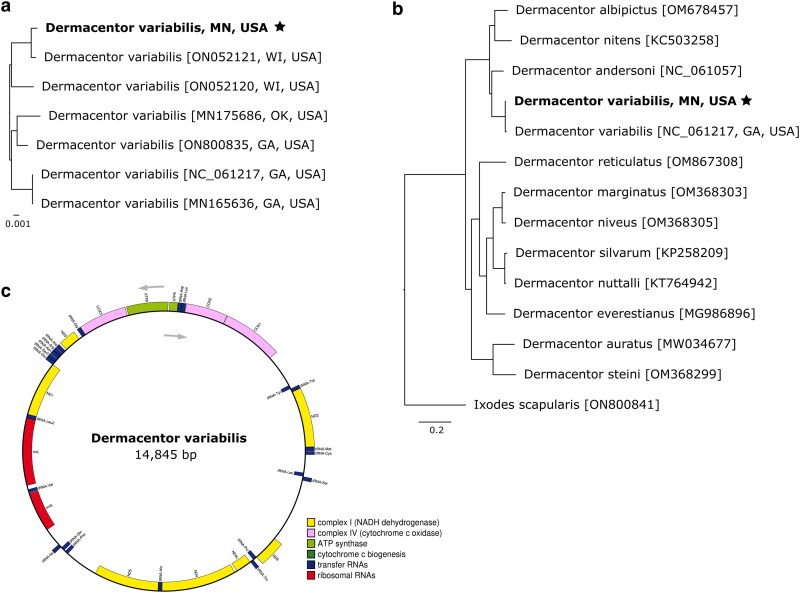
Phylogenetic relationships between *Dermacentor* species were reconstructed using bootstrapped maximum likelihood comparisons of our assembled mitogenome to existing hard tick reference mitogenomes. Phylogenies were created using the entire mitochondrial genomes. a) depicts relatedness among *D. variabilis* mitogenomes from throughout the United States, and b) displays relationships between all available *Dermacentor* mitogenomes, rooted with *I. scapularis* as the outgroup. c) displays the circular mitogenome with annotations.

The mitochondrial genome was extracted from the assembly and characterized with MitoHiFi, a tool explicitly designed for identifying and annotating mitochondrial contigs from long-read sequencing data (Uliano-Silva *et al*. 2023). The mitogenome assembly is a single circular contig spanning 14,845 bp, 6–8 bp longer than reference mitogenomes from various *D. variabilis* isolates on NCBI. The additional base pairs present in our mitogenome may stem from either homopolymer errors, a known limitation of nanopore sequencing, or true indels in our organism. Our mitogenome contained 22 tRNA genes, 13 protein-coding genes, and 2 rRNA genes consistent with the ancestral hard tick configuration (Wang *et al*. 2019; Fig. 2c), clustering monophyletically with other *D. variabilis* collected from the Upper Midwest (Fig. 2a). Although our mitogenome clustered with other isolates from the same geographical region, all *D. variabilis* mitogenomes were highly similar (>98.9%) over their entire length. Congeneric phylogenetic comparison supported previous analyses, clustering our mitogenome with the *D. variabilis* reference mitogenome (NC_061217), nested within a clade of *Dermacentor* spp. from the Americas, and distinct from closely related European and Asian *Dermacentor* spp. (Reynolds *et al*. 2023; Fig. 2b). The observed discordance between mitochondrial and nuclear phylogenies among *D. variabilis*, *D. andersoni*, and *D. albipictus* may reflect different evolutionary histories, introgression, incomplete lineage sorting, or other demographic processes. Prior work has noted conflicting nuclear and mitochondrial species trees, suggesting a polytomous relationship among these taxa (Lado and Klompen 2019; Leo *et al*. 2010; Reynolds *et al*. 2022).

### Repetitive DNA

Tick repeats are not well characterized in the public Dfam database, limiting our understanding of repetitive element content in the Ixodidae. To address this gap, we leveraged RepeatModeler2 to curate a custom database of de novo repeats from our draft assembly. We subsequently used RepeatMasker to mask these repeats in our final assembly. RepeatModeler discovered 4,372 distinct repeat families, 58% categorized as unknown. This finding is consistent with previous reports, which also found that more than 45% of repeats in the *I. scapularis* genome were unclassified (De *et al*. 2023). When we applied RepeatMasker to our assembly, it classified 61% of the genome as repetitive, with 32% of this content consisting of retroelements, predominately long interspersed nuclear elements (LINEs) and long terminal repeat (LTR) elements (Table 3). Only 2.5% of repetitive content was categorized as DNA transposons, in stark contrast to other hematophagous insects, such as tsetse flies, where DNA transposons comprise a substantial fraction of repetitive DNA (Attardo *et al*. 2019). Further, a considerable portion of the repetitive content (24%) remained unclassified, underscoring the complexity of transposable element (TE) dynamics in organisms with genomes dominated by selfish genetic elements. These unclassified repeats highlight the need for further investigation into the nature and role of TEs in tick genomes, which could provide insights into their evolution and genomic plasticity.

**Table 3.** Repetitive DNA content for the reported *Dermacentor* variabilis genome assembly.

RepeatMasker^a^	*D. variabilis* (%)
Retroelements	32.24
SINEs	0.7
LINEs	14.62
L2/CR1/Rex	4.03
R1/LOA/Jockey	5.7
RTE/Bov-B	2.29
LTR elements	16.91
Gypsy/DIRS1	14.94
DNA transposons	2.48
Tc1-IS630-Pogo	1.09
Unclassified	24.67
Total interspersed repeats	59.41
Simple repeats	1.25
Overall masked	61.1

^a^Only categories > 1% genomic content.

We applied our custom database of *D. variabilis* repeat families to mask 3 other published *Dermacentor* genomes. Our assembly shared a similar repeat content level with *D. albipictus* (59.64%), while *D. andersoni* (62.02%) and *D. silvarum* (54.27%) exhibited higher and lower levels, respectively. The lower repetitive content in our estimate of the *D. silvarum* genome compared to Jia *et al*. (2020) likely results from differences in the repetitive element databases used (Repbase vs RepeatModeler). Repeat content in the *D. variabilis* genome aligns with other metastriate tick genomes, such as *R. sanguineus* (61.6%) and *Haemaphysalis longicornis* (59.3%), but differs from prostriate ticks like *Ixodes scapularis* (69%) and *Ixodes ricinus* (73%), despite belonging to the same family (De *et al*. 2023; Ronai *et al*. 2024 ). These findings reinforce the phylogenetic relationships displayed in Figs. 1 and 2. Lineage-specific repetitive element content, driven by variations in TE activity, genome defense mechanisms (e.g. piRNA clusters), and evolutionary pressures, is evident across arthropod taxa (Petersen *et al*. 2019 ). Considering that TEs comprise the majority of tick genomes, understanding the forces shaping TE dynamics in hard ticks remains a priority. Detailed information is available in Supplementary File 2.

### Gene annotation

Putative gene predictions were completed using the GeMoMa annotation tool. GeMoMa predicts gene models utilizing existing gene models in related reference species (Keilwagen *et al*. 2019). We used the *I. scapularis* annotation (ASM1692078v2) as a reference, as it remains the most robust gene model among hard ticks. We detected 21,722 putative genes in our genome assembly. The protein BUSCO score was 80.88% complete (71.20% single copy and 9.68% duplicates), indicating a high level of completeness and reliability in the annotated genome. However, some regions may still contain fragmented or missing orthologs. Our genome annotation would benefit from detailed RNA-seq data and future methodological refinement. Annotations are available in Supplementary File 3.

### DNA methylation

We observed a genome-wide DNA methylation level of 9.9% in *D. variabilis*. Previous studies on other metastriate ticks, such as *D. silvarum* and *H. longicornis*, have characterized DNA methyltransferases, i.e. enzymes responsible for adding and maintaining methyl marks. Still, this study represents the first report of genome-wide methylation in metastriate ticks (Agwunobi *et al*. 2021).

**Fig. 3.**
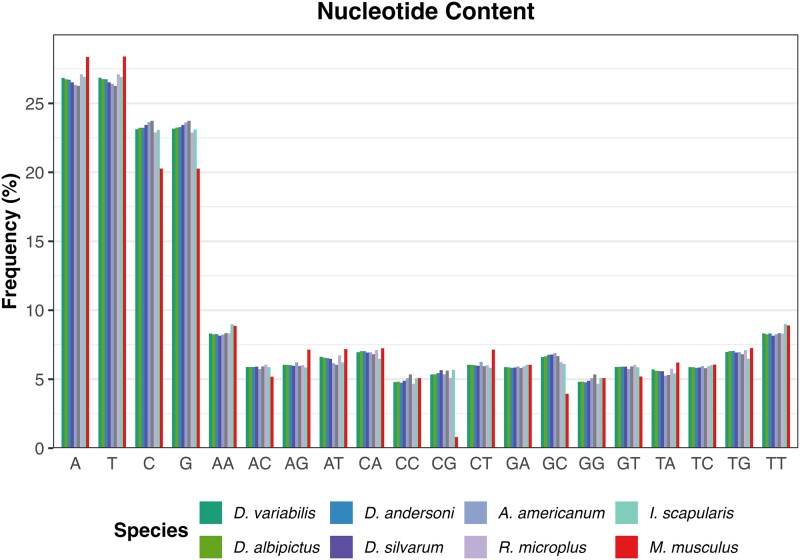
Distribution of nucleotide content comparing available hard tick nuclear genomes and *Mus musculus*, the house mouse.

The CpG dinucleotide composition of the *D. variabilis* genome suggests balanced transition rates compared to other Metastriate and Prostriate genomes, in contrast to the biased CpG depletion observed in the *Mus musculus* genome (mm39; Fig. 3). Mammalian genomes, with global methylation levels exceeding 70%, exhibit accelerated loss of CpG dinucleotides—a pattern not evident in tick genomes. In arthropods like ticks, methylation may contribute to phenotypic variation, particularly in contexts with limited genotypic variation. Recent studies highlight the role of epigenetic mechanisms in hard tick cold tolerance, reproduction, fecundity, and overall fitness (De *et al*. 2023; Nwanade *et al*. 2022; Yu *et al*. 2024). Future research should focus on elucidating the genomic distribution, spatial organization, and functional significance of methylation in ticks.

### Symbiont identification

**Fig. 4.**
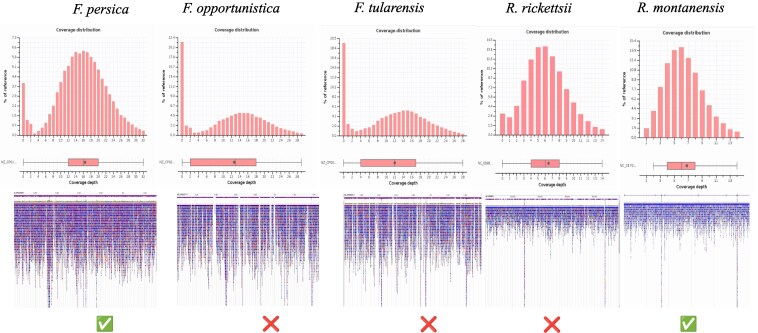
Alignment of mapped reads to the pathogenic and nonpathogenic (endosymbiont) reference genomes. Histograms display the coverage distribution for mapped reads, boxplots display the depth of coverage, and the genome viewer displays mapped read distribution across the reference genomes.

The American dog tick is a potential vector for Tularemia (*F. tularensis*) and Rocky Mountain Spotted Fever (*R. rickettsii*). All reads were aligned to potential reference genomes to confirm the presence of any symbionts, and breadth and depth were calculated. Contig BLAST hits matched to *Francisella persica*, *Francisella opportunistica*, and *F. tularensis*, which were chosen for alignment. The depth and breadth of mapped reads are displayed in Table 4 and Fig. 4. Mapped reads demonstrated the highest breadth and depth of mapping to *F. persica*, indicating the presence of only this nonpathogenic endosymbiont.

**Table 4.** Read alignment statistics for pathogenic and nonpathogenic microorganism reads extracted from the *Dermacentor variabilis* raw sequence data.

Target	Contigs	Avg. depth (×)	Reference (bp)	Length > 3× (bp)	Breadth (>3×) (%)	Present
*Francisella persica*	16	20.46	1,516,676	1,457,338	96.09	Yes
*Francisella opportunistica*	31	18.78	1,839,233	1,443,153	78.46	No
*Francisella tularensis*	10	18.47	1,870,206	1,503,758	80.41	No
*Rickettsia rickettsii*	0	6.5	1,257,710	1,216,534	96.73	Yes

Although no contigs were initially identified as *R. rickettsii*, we attempted to align reads to *Rickettsia montanensis* to determine its presence or absence, as it has been detected in *D. variabilis* ticks from Minnesota (Hecht *et al*. 2019). We identified reads mapping to 99% of the reference genome but at a lower depth than the *Francisella*-like endosymbiont, suggesting comparatively low levels of *Rickettsia* in *D. variabilis*. Intriguingly, we could not assemble contigs for this species, preventing definitive identification when using the kraken2 standard library. Difficulty in re-constructing the reads mapping to this genome may have arisen from its inherent lower cell count, inevitably leading to lower genome coverage. This affirms previous reports of *R. montanensis* presence in Minnesota and should be monitored, considering its association with clinical and subclinical symptoms in humans (Vincent and Hulstrand 2022).

## Conclusions

Our study presents the first whole-genome assembly for *D. variabilis*, the American dog tick, a hard tick widely distributed across North America. Our assembly achieved a BUSCO completeness score comparable to other publicly available *Dermacentor* genomes, underlining the utility of nanopore sequencing for generating high-quality tick genomes. Beyond its value for understanding tick biology and evolution, this assembly facilitates improved detection of tick-associated microbes, including endosymbionts and potential pathogens.

Amplification-free unbiased detection and identification of tick endosymbiont species within ticks remains challenging, owing to their complex evolutionary histories and low copy number compared to mitochondrial DNA. Nanopore sequencing allowed us to distinguish a *Francisella*-like endosymbiont, mapping more closely to *F. persica* than *F. tularensis*, using read mapping statistics (e.g. breadth and depth) to avoid false positives commonly encountered with PCR-based identification (Kugeler *et al*. 2005). Moreover, we detected *R. montanensis* through total read alignment to its reference genome despite an inability to assemble contigs for this species. These findings underscore the need to refine bioinformatic tools for long-read sequence data to better distinguish pathogenic from nonpathogenic bacterial DNA in ticks and highlights the importance of broadening genomic resources to improve our understanding of tick-microbe molecular interactions.

Expanding genomic resources for medically relevant arthropods is critical for uncovering evolutionary processes underlying vectorial capacity, migration, and life-history traits. Ticks, second only to mosquitoes in global vector significance, represent an expanding area for genomic exploration. Sequencing of hard tick genomes has provided key insights into genome biology, permitting comparative analysis between hard tick groups (i.e. Prostriata and Metastriata) that can reveal lineage-specific evolution of gene families, hematophagy, and ecological adaptations (Cerqueira De Araujo *et al*. 2025; De *et al*. 2023; Jia *et al*. 2020). Despite over 450 documented metastriate species, few genome assemblies are available on NCBI. Our *D. variabilis* genome helps close this gap, offering a critical resource for comparative analyses of tick genetic diversity, evolutionary history, and population genetics as ticks expand into new geographic areas and pose emerging zoonotic threats.

## Supplementary Material

jkaf130_Supplementary_Data

## Data Availability

Supplementary File 1 provides detailed descriptions of the computational steps outlined in the methods. Supplementary File 2 contains the transposon family database generated from RepeatModeler2. Supplementary File 3 contains the gene annotations in GFF format. Sequence data for the whole genome and mitogenome assemblies are deposited in GenBank with the accession number JBLSAA000000000. The assembly is available via the NCBI Genome archive at PRJNA1224226, BioSample SAMN46853854; the raw reads are available in the Sequence Read Archive under the same accession. Supplemental material available at G3 online.
