# Supplementary material for: The genome of the American dog tick (Dermacentor variabilis)
Source: G3 (Bethesda). 2025 Jun 9;15(8):jkaf130. doi: 10.1093/g3journal/jkaf130 (PMC12341941; doi:10.1093/g3journal/jkaf130)
Supplement: jkaf130_Supplementary_Data [file jkaf130_supplementary_data.zip › Supplemental_Material_Legends_G3-2025-405935.docx]

**Supplementary Files**

**Supplementary File S1:** Supplementary methods

**Supplementary File S2:** Transposon family database

**Supplementary File S3:** Annotations in GFF format

**Supplementary File S4:** BlobTools results

**Supplementary File S5:** Gene annotations
