# Supplementary material for: The genome of the American dog tick (Dermacentor variabilis)
Source: G3 (Bethesda). 2025 Jun 9;15(8):jkaf130. doi: 10.1093/g3journal/jkaf130 (PMC12341941; doi:10.1093/g3journal/jkaf130)
Supplement: jkaf130_Supplementary_Data [file jkaf130_supplementary_data.zip › Supplementary_File_S1_G3-2025-405935.html]

Dog tick genome


# Dog tick genome

#### chris faulk

#### 2025-02-14

## Project

Sequencing of samples from dog tick for genome assembly. Samples were
sequenced on the P2 Solo on R10.4.1 flowcells.

## Basecalling

Basecalling was performed in minknow during sequencing using the
dna\_r10.4.1\_e8.2\_400bps\_sup@v4.3.0 model. Re-basecalled with
dna\_r10.4.1\_e8.2\_400bps\_sup@v5.0.0.

```
# Posthoc basecalling
# Model dna_r10.4.1_e8.2_400bps_sup@v5.0.1, all cytosines
~/Desktop/dorado-0.8.2-linux-x64/bin/dorado basecaller sup,5mC_5hmC <input_directory> -r --min-qscore 10 > <output_file>.mod.bam
```

## Read QC and filtering

```
# Stats on read quality
samtools fastq file.bam | seqkit stats -a -
```

## Assembly flye

```
# Use flye 2.9.5

# Slurm file
#!/bin/bash -l
#SBATCH -A faulkc
#SBATCH --time=24:00:00
#SBATCH -p msibigmem
#SBATCH --ntasks=128
#SBATCH --mem=1995g
#SBATCH --mail-type=ALL
#SBATCH --mail-user=cfaulk@umn.edu

cd /home/faulkc/cfaulk/dogtick
flye --nano-hq Dog_tick_combined.fastq --out-dir flye-hq.allreads --threads 128
```

## Foreign Contaminant Screen

```
# Required prior to NIH ubmission.
# FCS-adapt removes adapter and vector sequences

# Install
curl -LO https://github.com/ncbi/fcs/raw/main/dist/run_fcsadaptor.sh
chmod 755 run_fcsadaptor.sh
curl https://ftp.ncbi.nlm.nih.gov/genomes/TOOLS/FCS/releases/latest/fcs-adaptor.sif -Lo fcs-adaptor.sif

# Run
sudo ./run_fcsadaptor.sh --fasta-input ../assembly.fasta --output-dir . --euk --container-engine singularity --image fcs-adaptor.sif

# Clean the genome (26 bp dropped)
curl -LO https://github.com/ncbi/fcs/raw/main/dist/fcs.py
cat assembly.fasta | sudo python3 fcs.py clean genome --action-report ./fcs_adaptor_report.txt --output clean.fasta --contam-fasta-out contam.fasta
```

## Purge\_dups

Purge\_dups was run to remove haplotigs and contig overlaps in a de
novo assembly based on read depth.

```
# Install
git clone https://github.com/dfguan/purge_dups.git
cd purge_dups/src && make -j 32

# Align the data to generate paf files
# minimap2 -x map-ont consensus.fasta $i | gzip -c - > $i.paf.gz
minimap2 -I 4G -x map-ont -t 32 ../clean.fasta ../../../Dog_tick_combined.fastq.gz | pigz > dogtick-p1.paf.gz

# Produce stats and cutoffs file
./purge_dups/bin/pbcstat dogtick-p1.paf.gz
./purge_dups/bin/calcuts PB.stat > cutoffs 2>calcults.log

# Split consensus and self-align
./purge_dups/bin/split_fa clean.fasta > clean.split.fa
minimap2 -xasm5 -DP clean.split.fa clean.split.fa | gzip -c - > clean.split.self.paf.gz

# Purge dups and haplotigs
./purge_dups/bin/purge_dups -2 -T cutoffs -c PB.base.cov clean.split.self.paf.gz > dups.bed 2> purge_dups.log

# Get purged primary and haplotigs
./purge_dups/bin/get_seqs -e dups.bed clean.fasta

# Generate histogram
./purge_dups/scripts/hist_plot.py -c cutoffs PB.stat PB.base.png
```

## Scaffolding

```
# Install ntLink for scaffolding with gap-filling
mamba install -c bioconda -c conda-forge ntlink

# Run with 3 rounds
mkdir ntlink-purged
cd ntlink-purged
ln -s ../purged.fa
ln -s ../../../../Dog_tick_combined.1k.fastq.gz 
ntLink_rounds run_rounds_gaps target=purged.fa reads=Dog_tick_combined.1k.fastq.gz k=32 w=100 t=5 rounds=6

# Split sequences by N to remove unfilled gaps
awk '/^>/ {print $0; next} {gsub(/[nN]+/, "\n>contig_subseq" ++i "\n"); print}' purged.fa.k32.w100.z1000.ntLink.6rounds.fa > purged-linked-nogaps.fa
```

## QC and BUSCO

```
# Read stats
# Assembly-stats
https://github.com/sanger-pathogens/assembly-stats

# Compleasm
mamba create -n compleasm -c conda-forge -c bioconda compleasm
mamba activate compleasm
compleasm run -t 32 -l arachnida -L ~/Desktop/genomes/mb_downloads -a assembly.fasta -o compleasm-your-name

# BUSCO v5.8.2
mamba create -n busco -c conda-forge -c bioconda busco=5.8.2
mamba activate busco
busco -i purged-linked-nogaps.fa -o busco -m genome --lineage arachnida_odb10 -c 32
```

## Assembly Evaluation with Quast

Quast was used to evaluate the assembly.

```
# Install
sudo apt-get update && sudo apt-get install -y pkg-config libfreetype6-dev libpng-dev python3-matplotlib
wget https://github.com/ablab/quast/releases/download/quast_5.3.0/quast-5.3.0.tar.gz

# Run
./quast-5.3.0/quast.py --large -t 32 -o quast-results-purgedlinkednogaps purged-linked-nogaps.fa
```

## MitoHiFi

```
# Pull docker container
sudo docker pull ghcr.io/marcelauliano/mitohifi:master

# Run through singularity
singularity shell --bind /home/ansc8520/dogtick/MitoHiFi/:/MitoHiFi docker://ghcr.io/marcelauliano/mitohifi:master mitohifi.py -h
Singularity> cd MitoHiFi
Singularity> findMitoReference.py --species "Dermacentor silvarum" --outfolder . --min_length 14000
Singularity> mitohifi.py -r Dog_tick_combined.2k.fastq.gz -f NC_026552.1.fasta -g NC_026552.1.gb -t 32 -o 5

# The "potential contigs" directory lists the contig containing mtDNA (contig_12841).
# Annotations were saved for submission to NCBI Assembly.
```

## Mitochondrial phylogeny

```
# Install MAFFT and iqtree2

# Align
mafft --auto muriqui_mitogenomes.fa > muriqui_mitogenomes.aln

# Tree
iqtree2 -s muriqui_mitogenomes.aln

# Visualize with FigTree
```

## Repeats

### RepeatModeler

```
# Install Singularity
wget https://github.com/sylabs/singularity/releases/download/v4.1.4/singularity-ce_4.1.4-jammy_amd64.deb

# Install DFAM TEtools
curl -sSLO https://github.com/Dfam-consortium/TETools/raw/master/dfam-tetools.sh
chmod +x dfam-tetools.sh
./dfam-tetools.sh

# Build database
BuildDatabase -name rm-dogtick purged-linked-nogaps.fa 

# Run RepeatModeler
RepeatModeler -database rm-dogtick -LTRStruct -threads 32
```

### RepeatMasker

```
# Default library from DFAM
RepeatMasker -spec arachnids -s -pa 32 -xsmall -gff -e rmblast purged-linked-nogaps.fa

# Custom library created with RepeatModeler2
RepeatMasker -lib rm-dogtick-families.fa -s -pa 32 -xsmall -gff -e rmblast purged-linked-nogaps.fa
```

## Align reads to final genomes

```
# Create modmapped bam
~/Desktop/dorado-0.8.3-linux-x64/bin/dorado aligner purged-linked-nogaps.fa dogtick_total.mod.bam > dogtick_total.modmapped.bam
```

## Methylation

```
# Modkit
~/Desktop/modkit_0.4.1/modkit pileup --ref purged-linked-nogaps.fa --cpg dogtick_total.modmapped.bam dogtick_total.modmapped.bam.bed

# Summarize
for i in *.bed; do  awk -v file="$i" '$4=="m" {can+=$13; mod+=$12; oth+=$14; valid+=$10} END{print file "\tCpG canonical " (can/valid) "\tCpG methyl " (mod/valid) "\tCpG hydroxy " (oth/valid)}' $i >> methylation-summary.txt; done
```

## GeMoMa Gene Annotation

Wiki

```
# Install dependency `mmseq`:
mamba install -c conda-forge -c bioconda mmseqs2

Installed version 1.8 [@Keilwagen:2018:GeMoMa_RNAseq; @Keilwagen:2016:GeMoMa] by downloading `.zip` file from [website](https://www.jstacs.de/index.php/GeMoMa#Requirements)

# Run vs Ixodes scapularis annotations. 
# Genome assembly ASM1692078v2

java -Xmx50g -jar GeMoMa/GeMoMa-1.9.jar CLI GeMoMaPipeline threads=32 outdir=annotation GeMoMa.Score=ReAlign AnnotationFinalizer.r=NO o=true t=../final-assembly/purged-linked-nogaps.fa i=I_scapularis a=GCF_016920785.2_ASM1692078v2_genomic.gff.gz g=GCF_016920785.2_ASM1692078v2_genomic.fna.gz

# Busco on protein mode:
compleasm download arachnida
compleasm run -t 32 -l arachnida -L ~/Desktop/genomes/mb_downloads -p predicted_proteins.fasta -o compleasm-proteins
```

## Blobtools

### Identify contigs by NCBI Blast.

```
# First download the core_nt database from NCBI.
update_blastdb.pl --decompress core_nt

# The following blast query will provide correct input format for blobtools.
blastn -query ../final-assembly/purged-linked-nogaps.fa -task megablast -db core_nt_blast_db/core_nt -outfmt '6 qseqid staxids bitscore std sscinames sskingdoms stitle' -culling_limit 5 -num_threads 32 -evalue 1e-3 -out purged-linked-nogaps.vs.nt.1e3.megablast.core_nt.out
```

### Examine assembly with Blobtoolkit

```
# Filter the assembly for bacterial reads using BlobTools2.Generate a coverage
samtools coverage ../dogtick_total.modmapped.bam > dogtick_total.modmapped.sorted.txt

#Create and add data to the blobdir: 
blobtools create --fasta ../final-assembly/purged-linked-nogaps.fa --meta dogtick.yaml --taxid 34621 --taxdump taxdump/ dogtick
blobtools add --hits purged-linked-nogaps.vs.nt.1e3.megablast.core_nt.out --taxdump taxdump/ dogtick 
blobtools add --text dogtick_total.modmapped.sorted.txt --text-header --text-cols '#rname=identifier,meandepth=dogtick_reads_cov' dogtick 
blobtools add --key plot.y=dogtick_reads_cov dogtick 

# View blobplots in a browser:
blobtools view --local --interactive dogtick 

http://localhost:8007/view/dogtick/dataset/dogtick/blob
```

#### Manually remove contaminants

Look at the csv made by blobtools and keep only “arthropod” and
“no-hit” contigs. Remove them using seqkit and save as
purged-linked-nogaps-filtered.fa. Examine and remove contigs greater
than 1000X and less than 1X.

`seqkit grep -v -f filter_contigs.txt input.fasta -o output.fasta`

## Microbiome

```
# Filter for reads that don't align to the tick genome.
samtools view -b -f 4 mapped.sorted.bam > dogtick_non-host.mapped.bam
samtools fastq dogtick_non-host.mapped.bam > dogtick_non-host.fastq
```

### Mapping to target genomes

```
# Minimap the francisella genomes against all the reads and filter for only matching hits
minimap2 -ax map-ont -t 32 francisella-spp.fna ../dogtick_total.fq.gz | samtools view -b -F 4 - | samtools sort -@ 32 > dogtick_total-francisella-spp.bam

samtools fastq dogtick_total-francisella-spp.bam > dogtick_total-francisella-spp.fq

# Assemble reads
flye --nano-raw dogtick_total-francisella-spp.fq --out-dir flye_assembly --genome-size 3m --threads 32

# Get breadth of coverage (SAMtools)
# create samtools index
samtools index mapping_result_sorted.bam

# get total number of bases covered at MIN_COVERAGE_DEPTH or higher
samtools mpileup mapping_result_sorted.bam | awk -v X="${MIN_COVERAGE_DEPTH}" '$4>=X' | wc -l

# get length of reference genome
seqkit stats file.fa
```

###
